# Protective factors for suicidal ideation: a prospective study from adolescence to adulthood

**DOI:** 10.1007/s00787-024-02379-w

**Published:** 2024-02-14

**Authors:** Victoria Bakken, Stian Lydersen, Norbert Skokauskas, Anne Mari Sund, Jannike Kaasbøll

**Affiliations:** 1 Regional Centre for Child and Youth Mental Health and Child Welfare (RKBU), Department of Mental Health, Faculty of Medicine and Health Sciences, Norwegian University of Science and Technology (NTNU), Trondheim, Norway; 2 Department of Health Research, SINTEF, Trondheim, Norway

**Keywords:** Suicide ideation, Protective factors, Adolescence, Longitudinal studies, Cohort study

## Abstract

Adolescent suicidality is associated with negative outcomes in adulthood. Suicide prevention has traditionally focused on identifying risk factors, yet suicide rates have remained stable. With suicidality often going undetected—especially suicidal ideation, further knowledge about protective factors is needed. The main objective of this study was to investigate potential protective factors for suicidal ideation from adolescence to adulthood. The study employed longitudinal population survey data, “Youth and Mental Health Study” consisting of self-reports at two-time points (mean age 14.9, *SD* = 0.6 and 27.2, *SD* = 0.6) (*n* = 2423 and *n* = 1198). Protective factors (at individual, social and environmental level) were selected based on a priori knowledge. Internal consistency of scales was analyzed using McDonald’s omega. We used a linear mixed model with suicidal ideation as the dependent variable, time-points, a protective factor variable and their interaction as covariates, and individual participant as random effects. We adjusted for sex and also conducted separate analyses for males and females. The Benjamini–Hochberg procedure was used to adjust *p*-values for multiple hypotheses. Investigated protective factors were associated with temporal change in suicidal ideation (significant interactions). For both sexes, less emotion-orientated coping, higher self-perception scores, greater levels of physical activity and higher school wellbeing/connectedness were protective factors for suicidal ideations. Secure attachment and higher family function were protective factors for females only. The effects in adolescence were mostly maintained in adulthood. In this study, several protective factors for suicidal ideation persisted into adulthood, with distinct differences between males and females.

**Supplementary Information:**

The online version contains supplementary material available at 10.1007/s00787-024-02379-w.

## Introduction

Suicide is globally the fourth leading cause of death among 15–19-year-olds [1]. Suicidality at a young age is not only associated with increased suicide mortality but also with adverse outcomes later in life [2–4]. The suicide rate among adolescent males is higher than in females [5, 6], and this difference persists into adulthood [7]. A history of attempted suicide, mental health problems or disorders, psychosocial risk factors (i.e. adverse life events, trauma), and minority background are among the strongest predictors of suicide [6, 8]. Suicidal ideations (SI) means to have thoughts or ideas (i.e. contemplations, preoccupations or wishes) about suicide and death [9] and is an important aspect of considering suicide risk [10]. Although most adolescents experiencing SI will not act upon their thoughts [11], SI is a robust predictor of suicide attempts and deaths at all ages [12]. SI is more common than suicidal behaviours (e.g. attempts), but is less likely to be detected by those close to adolescents (e.g. parents) or professionals [13, 14]. In adolescence, females report SI more often than males, but in adulthood male reports of SI increase [15]. Despite longstanding clinical -and research attention, increased public awareness, as well as governmental support, global suicide rates have remained stable for decades [16], and the prevalence of SI and suicidal behaviour has increased worldwide over the past 15 years [17, 18]. Previous suicide research and prevention efforts have predominantly focused on identifying risk factors or “warning signs” while protective factors that may reduce SI, and therefore possibly prevent suicide, have largely been unexplored [19].

Protective factors are defined as factors at individual, social and environmental level that lessen the disadvantageous outcome effects of adversity [20]. As the overall conceptual framework for the study, the protective factor model refers to processes in which promotive factors that moderate negative effects of risks for predicting negative outcomes. In this model, promotive factors are called protective factors to distinguish them from promotive factors that only compensate for risk exposure [21]. In the context of suicidality, protective factors decrease risk while promoting health and subjective wellbeing [22]. Due to the limited evidence, studies that cover SI and suicidal behaviours (i.e. attempts) should be considered to understand protective factors for suicidality. Protective factors are closely linked to resilience—an individual’s positive adaptions when faced with adversity or life challenges [23]. Likewise, individual coping strategies involve adaptive and sometimes protective processes when stressors are encountered [24]. A cross-sectional study [25] found that task-oriented coping styles reduced SI, while emotional-oriented and avoidant styles increased SI when depression was present. Emotion-orientated coping (i.e. an individual's efforts to reduce stress through emotional responses such as feelings of hopelessness, and blame of self or others [26]) has in particular been linked to SI and suicidal manifestations in both sexes [27]. Furthermore, a systematic literature review [28] and cross-sectional studies [29, 30], indicate that self-perceived competencies (i.e., self-esteem, self-worth, and appearance) are related to less SI in adolescence. Healthy lifestyles, such as physical activity are also associated with less SI among adults, however, in adolescents, findings are ambiguous [31]. Albeit, undertaking extra-curricular activities have been found to protect adolescents from SI and were interconnected with higher self-esteem [32]. As most of these studies were cross-sectional, causal relationships could not be established. According to previous studies [5–7] and a recent systematic review and meta-analysis [33], it is well established that there are sex differences in risk factors for suicidality. However, few investigate sex differences in protective factors. Hence, more research is needed to explore this gap in research.

Family and social connectedness have been suggested to be protective factors for suicidality [30]. Secure attachment is a significant and core component of resilience, according to a recent systematic review [34]. A longitudinal cohort study [35] identified family attachment as a protective factor against suicide attempts. Results from a cross-sectional study suggested that better family functioning (i.e. communication and emotional support) reduced the levels of SI among adolescents [36]. The literature on peer support in relation to suicidality is mixed [37]. According to a literature review, positive peer support protects against several undesirable mental health outcomes, including SI, in adolescence. According to a longitudinal study, social relationships can be both harmful and protective, depending on how they influence individuals [39]. A cohort study [40] found no protective effects for family cohesion, social support, high self-esteem, and mastery on adolescent suicide attempts. At the environmental level, higher school connectedness was found to reduce SI and suicidal behaviour [41] in both general and high-risk adolescent populations. In addition, knowing where to seek help [42], religion [19], cultural connectedness [43] and restricted access to lethal means [44] have also been reported to prevent suicides. Compared to adolescents with low or high socioeconomic status (SES), high-middle SES adolescents have lower odds of SI [45].

In brief, there is limited and sometimes ambiguous evidence on protective factors for SI. The majority of studies have predominantly focused on suicidal behaviour, whereby SI often is included as a suicidal behaviour rather than explicitly investigated [46], and knowledge of resilience and growth following adversity mostly derives from research in clinical settings or in vulnerable groups [47]. Most striking is the lack of longitudinal studies on protective factors for SI among adolescents [33, 38]. The objective of this study was to investigate potential protective factors for SI from adolescence to young adulthood, with the following research question: *What are the protective factors at the individual, social and environmental level (coping strategies, self-perceptions, physical health, peer/parent attachments, family function, school, and SES) associated with SI reduction from adolescence to young adulthood*? *Are there sex differences?*

It is hypothesized that higher levels of protective factors during adolescence will be associated with reduced SI in adolescence, with long-term effects into adulthood which may differ between males and females.

## Methods

### Study design and participants

**Fig. 1:**
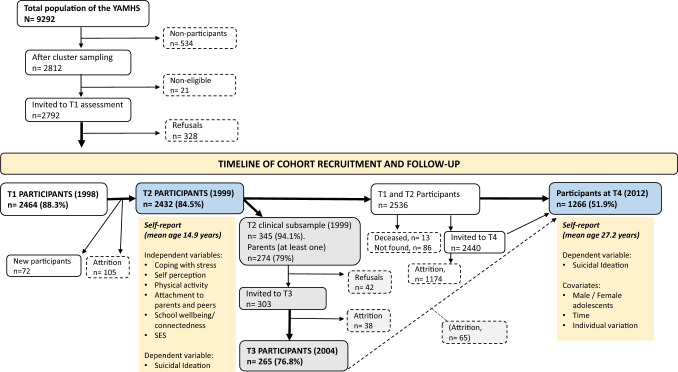
Timeline and procedure for cohort recruitment and follow-up in the Youth and Mental Health Study. T1 = Timepoint 1, T2 = Timepoint 2, etc. Data utilized in the current study are highlighted in blue at T2 and T4, along with additional listed information

Data stem from the population-based, prospective “Youth and Mental Health Study” (YAMHS) [48] (Fig. 1) which explored adolescent risk and resilience factors in relation to mental health, especially depressive conditions [48]. In total 9292 adolescents attended 8th and 9th grades in Trøndelag counties in central Norway in 1998, with most adolescents attending public schools (98.5%). Using proportional allocation, a representative sample of this population (2812 students, from 22 schools) was selected. Small schools (*n* = 534), i.e., those without a class each for every grade, were excluded. For the following reasons, 21 pupils (0.7%) were not eligible for the assessment: being ill on the day of the assessment, at an institution, not fluent in Norwegian, or recently arriving in Norway. A total of 2792 adolescents were eligible for the study. Parents and adolescents both received an invitation letter to participate in the study. Participants were not economically compensated, but could win a prize at each study wave (worth 8,000 (T1 and T2) and 10,000 (T4) Norwegian kroner). The YAMHS baseline data was collected in 1998 (T1) in two counties of central Norway from 2,464 adolescents (response rate 88.3%, mean age 13.7 (*SD* = 0.6) years, 50.8% female). Adolescents completed the self-report questionnaires during two consecutive school hours. The first follow-up was conducted in 1999 (T2) (*n* = 2,432, response rate of 87.1%, mean age 14.9 (*SD* = 0.6) years, 50.4% female). Clinical interviews were used to evaluate a subgroup at T2 (*n* = 345) and T3 in 2005 (not part of the present study). The most recent follow-up was conducted in 2012 (T4) (*n* = 1266, 51.9%, mean age 27.2 (*SD* = 0.6) years, 56.7% female). T4 questionnaires were completed online. The present study used data from the two timepoints T2 and T4. A total of 1160 had data on SI at both timepoints. As adolescents (T2) *n* = 2423 had complete data and as adults (T4), *n* = 1198 had complete data (49.4% of the T2 participants who also took part at T4).

### Suicidal Ideation from adolescence to adulthood: the outcome variable

Suicidal Ideation (SI) was measured by the mean score made up by five items. Four items were from the Mood and Feelings Questionnaire (MFQ), which assesses SI among other symptoms of depression in adolescents [49]. MFQ questions comprised of: “I thought that life was not worth living”, “I thought about death or dying”, “I thought my family would be better off without me”, and “I thought about killing myself”. One additional item from the Center for Epidemiologic Studies Depression Scale (CES-D) was included: “I would have killed myself if I had known a way of doing it”[50]. It was added to provide a more comprehensive picture of the concept of SI, which may also involve more passive thinking [51]. Respondents are asked whether descriptions in the questionnaire are 'true' (1), 'sometimes true' (2) or 'not true' (0) for them over the past two weeks.. A dichotomous variable was created, coding 0 as No SI, and 1 or 2 as Yes SI. The SI items of the MFQ have been validated in its performance both for detecting concurrent and predicting future SI [52]. The SI composite scale of five items previously used in the YAMHS has shown good reliability [51, 53]. The distribution of SI was highly skewed. In this study, the internal consistency of the SI scale was computed using McDonalds Omega, which is robust also for variables with skewed distributions [54]. Omega was 0.87, suggesting good internal consistency.

### Protective factors in adolescence: independent variables

Potential protective factors among those available in the YAMHS were selected based on a priori knowledge of individual assets and ecological resources identified by previous research [38]. In this study, these were categorized into three levels: Individual, Social and Environmental protective factors.

#### Individual factors

*Coping traits* were measured by the Coping Inventory for Stressful Situations (CISS). The CISS scale (1990 version) measures three coping dimensions based on theoretical and empirical evidence and has established robust psychometric properties [26, 55]. Responses are made up of a four-point Likert Scale whereby higher scores indicate a high level of the coping trait. The shortened scale has 17 items in total; Emotion-orientated (6 items), Task-orientated (5 items) and Avoidant coping (6 items). McDonald’s Omega had values of 0.79 for Emotion coping, 0.84 for Task coping and 0.64 for Avoidant coping -indicating good internal consistency for Emotion -and Task coping, but less so for Avoidant coping.

Adolescents’ *self-perception* was measured using the Norwegian-adapted version of the “Self-Perception Profile for Adolescents” (SPPA). The Norwegian version reduced the original nine subscales to seven after cultural adaption and removal of low-reliability scales [56]. The YAMHS selected three subscales, “Global Self-Worth”, “Physical Appearance” and “Social Competence”, making 15 questions in total. Responses consisted of a four-point Likert scale, whereby a higher score indicated positive self-perceptions. The scales had good internal consistency with the following Omega; 0.80 for Global Self-worth, 0.89 for Physical Appearance and 0.79 for Social Competence.

The level of *physical activity* was measured using a single item from the YAMHS questionnaire, which was modified by Paffenbarger et.al. [57]. Participants were asked to rank their level of activity using a five-point Likert scale. A higher score on this scale equates to being more physically active.

#### Social factors

*Attachment* was measured using a revised version of “Inventory of Parent and Peer Attachment” (IPPA), comprising separate scales for attachment to mother, father, peers [58]. The IPPA instrument assesses adolescent perceptions of cognitive and affective dimensions of their relationships and is scored using a five-point Likert scale. The parent scales are made up of 25 items each, and the peer scale was shortened to 9 items based on the removal of low-reliability items in earlier research constituting a unidimensional scale. High scores indicate secure attachment. IPPA has good psychometric value and good reliability [59, 60]. Total mean scores were calculated separately for the three scales in the present study. There was good internal consistency for attachment to parents with McDonald’s Omega equal to 0.91 for attachment to mother, 0.90 for attachment to father. However, lower internal consistency was found for attachment to peers with an Omega equal to 0.70.

To measure adolescent perceptions of their own *family functioning*, the subscale “General Functioning” from the McMaster Family Adjustment Device (FAD) was utilized [61]. This subscale comprised 12 items, with responses reported using a five-point Likert scale. Higher scores indicated better functioning within the family, according to adolescents who self-reported. The General Family Functioning scale had a McDonald’s Omega of 0.86, showing good internal consistency.

#### Environmental factors

The adolescent’s *connectedness and wellbeing at school* (class and teacher) were assessed with two separate items from scales developed specifically for the YAMHS [48], one item from the “Class Wellbeing Scale” – on wellbeing and connectedness to the school class, and the other item from the “ Teacher Support Scale” – on experienced support from the teacher(s). Responses were given on a four-point Likert Scale, whereby higher scores reflected better connectedness and wellbeing in a school setting.

The scores of participants’ *Socio-Economic Status (SES)* used in the present study were calculated at T1 in the YAMHS. The SES measure is based on adolescent self-report on their mother and father’s occupations. Responds included five categories as classified by ISCO-88; manual workers, primary industry, lower middle class, upper middle class and professional leader [62]. In the present study categories were coded on a five-point Likert Scale, whereby higher scores indicated higher SES.

### Statistical analysis

The internal consistency of scales was analyzed using McDonald’s omega. We used a linear mixed model with SI as a dependent variable, time (T4 versus T2), a protective factor and their interaction as covariates, and the individual participant as a random effect (one protective factor at a time). The linear mixed model included participants with data on at least one time point and gives unbiased results if data are missing at random (MAR). A complete case would have included fewer observations and would give unbiased results only under the more restrictive missing completely at random (MCAR) assumption [63]. The analyses were adjusted for sex and were also done separately for males and females. As the distribution of the dependent variable of SI was highly skewed, the robust variance estimator was used. We present the effects on SI using the upper and lower quartile scores of the protective factors. The Benjamini–Hochberg procedure was used to adjust *p*-values for multiple hypotheses to preserve the false discovery rate (FDR) for the 28 analyses (14 variables for females and for males). Adjusted *p*-values under *p* < 0.05 were regarded to represent statistical significance. Adjusted *p*-values and 95% confidence intervals (CI) were reported where relevant. A significant interaction means that the change in SI from T2 to T4 depends on the protective factor. McDonald’s omega was calculated in SPSS 28, Benjamini–Hochberg adjustment was conducted in R.Studio and the other analyses were in Stata 17.

## Results

### Characteristics of the sample

The study employed two data samples T2 (*N* = 2423, female *n* = 1222, male *n* = 1201, mean age: 14.9, *SD* = 0.6) and T4 (*N* = 1198, female *n* = 694, male *n* = 504 mean age: 27.2, *SD* = 0.6). The distribution of SES was as follows: professional leader (upper class) (10.9%, *n* = 132), upper middle class (32%, *n* = 407), lower middle class (11.8%, *n* = 150), primary industry worker (8.5%, *n* = 108), manual worker (32.6%, *n* = 413), and missing values (4.4%, *n* = 56). Self-reported SI showed that during adolescence (T2), females (*M* = 0.19, *SD* = 0.39) reported higher levels of SI than males (*M* = 0.09, *SD* = 0.27), while males in young adulthood (T4) showed higher mean scores (*M* = 0.12, *SD* = 0.31) (Online Resource 1).

### Predication of change—interactions between protective factors and suicidal ideation

As displayed in Table 1, for both sexes, the interactions were statistically significant (*p* < 05) for seven of the proposed protective variables on the outcome of SI (Emotion-orientated coping, Global self-worth, Physical appearance, Social competence and Level of physical activity, Class wellbeing/connectedness and Teacher support).

**Table 1:** Interactions between Protective Factors and Suicidal Ideation from Adolescence to Adulthood

**Protective factor**	**Sex**	**Estimated marginal mean of suicidal ideation**				**Interaction coefficient**			
		**T2**		**T4**					
		**Low**^a^	**High**^**b**^	**Low**	**High**	**Estimate**	**95% CI**		**Adjusted *p*-value**^**c**^
							**LL**	**UL**	
Emotion-orientated coping ^d^	Female	.044	.231	.077	.101	− .182	− .249	− .115	** < .001**
	Male	.047	.148	.110	.109	− .112	− .169	− .055	** < .001**
Task-orientated coping	Female	.225	.155	.107	.086	.049	− .014	.113	.161
	Male	.089	.077	.132	.086	− .034	− .084	.015	.210
Avoidant coping ^d^	Female	.185	.186	.094	.096	.001	− .080	.084	.965
	Male	.074	.098	.112	.106	− .049	− .129	.030	.260
Global self-worth	Female	.227	.026	.099	.083	.046	.035	.057	** < .001**
	Male	.171	.045	.143	.099	.020	.007	.033	**.005**
Physical appearance	Female	.244	.069	.102	.082	.025	.015	.035	** < .001**
	Male	.202	.056	.125	.109	.021	.010	.032	** < .001**
Social competence	Female	.234	.114	.109	.074	.021	.007	.034	**.004**
	Male	.139	.034	.123	.102	.021	.007	.035	**.005**
Level of physical activity	Female	.270	.098	.093	.078	.057	.032	.081	** < .001**
	Male	.164	.029	.131	.061	.035	.009	.062	**.011**
Attachment to mother	Female	.271	.090	.101	.087	.219	.158	.281	** < .001**
	Male	.123	.043	.128	.097	.063	− .005	.133	.107
Attachment to father	Female	.244	.092	.100	.093	.174	.116	.231	** < .001**
	Male	.122	.054	.134	.088	.026	− .035	.088	.446
Attachment to peers	Female	.120	.202	.117	.093	− .136	− .222	− .049	**.004**
	Male	.092	.084	.120	.098	− .016	− .070	.037	.576
General family functioning	Female	.254	.086	.100	.086	.229	.165	.292	** < .001**
	Male	.126	.045	.131	.094	.066	− .016	149	.153
Class wellbeing/connectedness	Female	.229	.118	.109	.078	.080	.030	.130	**.004**
	Male	.114	.037	.122	.100	.054	.015	.093	**.010**
Teacher support	Female	.190	.105	.099	.104	.091	.042	.139	** < .001**
	Male	.089	.042	.116	.128	.059	.028	.090	** < .001**
Socio-economic status	Female	.200	.156	.083	.094	.018	− .002	.038	.109
	Male	.086	.084	.098	.115	.006	− .012	.025	.556

*Note*: *T2* adolescents, *T4* adults, *LL* Lower limit, *UL* Upper Limit

^a^Low = Lower quartile scores

^b^High = Upper quartile scores

^c^Benjamini-Hochberg

^d^low scores = protective

**Fig. 2:**
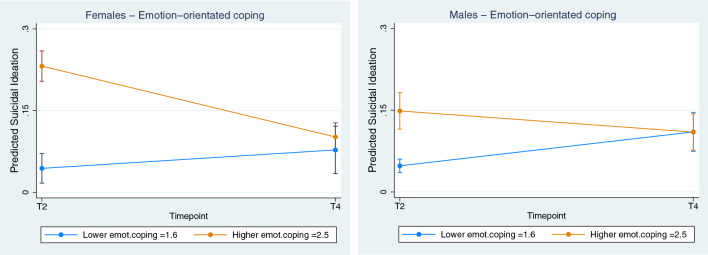
Individual—Effect of Coping orientation (Emotion-orientated) on Suicidal Ideation, Female vs Male**.** Lower (blue line) = lower quartile scores, Higher (orange line) = Upper quartile scores. Note, Low scores are in this case desirable/protective, high score not protective

More specifically, among scores from the Coping Inventory for Stressful Situations (CISS), it was only the subscale for Emotion-orientated coping (Fig. 2) that had a significant interaction—for both females (coefficient, -0.182; CI, -0.249 to -0.115, *p* < 0.001) and males (coefficient, -0.112; CI, -0.169 to -0.055, *p* < 0.001).

**Fig. 3:**
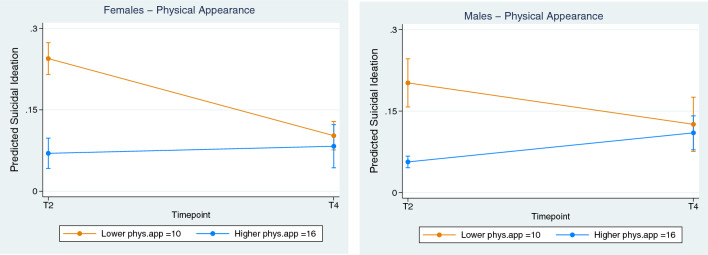
Individual—Effect of Self-Perception (Physical Appearance) on Suicidal Ideation**.** Female vs Male**.** Lower (orange line) = lower quartile scores, Higher (blue line) = Upper quartile scores

All three subscales (global self-worth, physical appearance, and social competence) within the “Self-Perception Profile for Adolescents” (SPPA) yielded significant interaction effects for both sexes. Physical appearance (Fig. 3) had the greatest effect, with high scores being associated with lower SI, almost identically for females (coefficient, 0.025; CI, 0.015 to 0.035, *p* < 0.001) and males (coefficient, 0.021; CI, 0.010 to 0.032, *p* < 0.001).

Also at an individual level, physical activity adolescents had a significant interaction. The strongest interaction was seen for females (coefficient, 0.057; CI, 0.032 to 0.081, *p* < 0.001). Highly active females had lower reported SI as adolescents and low levels were maintained into adulthood. The interaction was also significant for males (coefficient, 0.035; CI, 0.009 to 0.062 *p* = 011). However, despite low SI as adolescents, SI increased in adulthood for males.

**Fig. 4:**
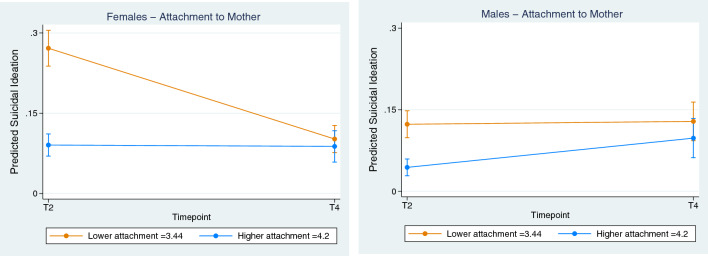
Social—Effect of Attachment (to Mother) on Suicidal Ideation. Female vs Male. Lower (orange line) = lower quartile scores, Higher (blue line) = Upper quartile scores. Note; Interaction effect only significant for females

Protective factors at the social level (Attachment to parents and peers, and family functioning) had a significant effect on SI for females only. Figure 4 displays the effect of Attachment to the mother, whereby higher attachment is associated with reduced and stable SI scores from adolescence to adulthood for females (coefficient, 0.219; CI, 0.158 to 0.281, *p* < 0.001). Similar effects were seen in attachment to the father. On the contrary, higher attachment to peers was the only proposed protective factor which instead was associated with increased SI in adolescent females.

**Fig. 5:**
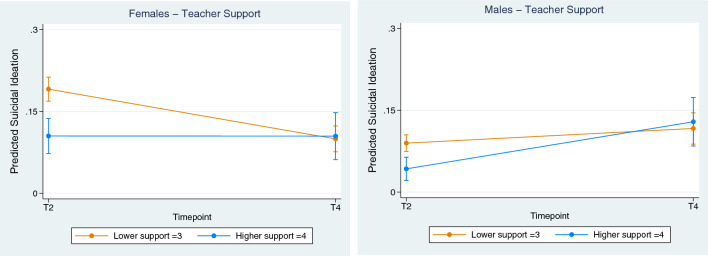
Environmental—Effect of School (Teacher Support) on Suicidal Ideation. Female vs Male**.** Lower (orange line) = lower quartile scores, Higher (blue line) = Upper quartile scores

Finally, the school environment had a significant interaction for both sexes. As displayed in Fig. 5, higher levels of self-reported teacher support were associated with low SI for females (coefficient, 0.091; CI, 0.042 to 0.139, *p* < 0.001) and males (coefficient, 0.059; CI, 0.028 to 0.090, *p* < 0.001). Figure 5 suggests a stable low SI outcome for females who received higher levels of teacher support as adolescents.

## Discussion

This study found that less emotion-orientated coping, positive self-perceptions (self-worth, social competence, and physical appearance), greater levels of physical activity and school wellbeing/connectedness were significant protective factors for SI in both adolescent sexes. This supports earlier findings on coping [25, 27], self-esteem and self-concept [29, 30], activity [32] and school connectedness [41]. However, task-oriented and avoidant coping styles, and socio-economic status had no significant interaction with SI for either sex in this study which contradicts some previous study findings [25, 45]. Sample differences may explain results that diverged from previous findings, i.e. clinical vs general, sample sizes, measurement instruments, sex and age differences. Adding to the existing literature, the results of this study indicated that most protective factors had a lasting stable or reductive effect into adulthood. Several studies involving protective factors and resilience are cross-sectional and conducted at one timepoint, often with vulnerable groups and with a primary focus on internal/individual factors [38, 47]. No previous study has investigated ecological protective factors longitudinally, with a population sample.

Attachment and family functioning were protective factors for SI in females only. Despite secure attachment’s significance across the lifespan and for developing resiliency [34] social protective factors can be temporarily changed in adolescence due to transitions (i.e. changes in school, relationships and family status). Females also seem to have a higher sensitivity to interpersonal and relational insecurities in the transition to adolescence [64] -which could make the attachment more critical as seen in this study. However, similarly to past research [39] negative effects of peer attachment were also present, with higher peer attachment increasing rather than decreasing SI in adolescent females. Regarding males, it is possible that protective factors at the social level have different functions and are not dependent on the level of attachment or family functioning. Societal and cultural norms around masculinity may discourage help-seeking and support from family and peers. Thus, research shows that males may benefit from social support through other arenas (i.e. work-environment, physical activity) [65]. It is, therefore, possible that males are better protected from suicidality in social contexts with opportunities to connect in environments outside closest attachments.

Sex differences were also evident with distinct patterns at the two timepoints. Females reported over twice the frequency of SI compared to males as adolescents. However, as SI tended to decrease overall in adulthood for females, male SI tended to increase despite the previous effect of protective factors during adolescence. The reduction of SI from adolescence to adulthood is especially pronounced for females who had initially lower scores on protective factors. These findings support notions of high SI among adolescent females [66], but also identified an increase in SI as males grow older [15]. Considering the known vulnerability factors in mental health and wellbeing in adolescent females, protective factors might be more impactful for males in adolescence. In addition, with both personal and interpersonal positive perceptions developing from adolescence to adulthood [67] these changes may have protective effects on females. Females have been found to have steady self-esteem growth, unaffected by other covariates, in their development from adolescence to adulthood [68]. Moreover, a growing body of research suggests males may be most vulnerable in early adulthood due to social expectations, roles, and responsibilities [69]. This may also contribute to the suicide mortality rates among males, which increases in mid-life and peaks in older age [7].

### Limitations

Some limitations should be noted about this study. Firstly, the sample showed a low representation of non-Norwegian ethnicities and little variation in socio-economic status [48]. Data collection took place in a homogeneous county, making the sample representative of the population. It does, however, limit international generalizability—including ethnicity, socioeconomic status, and cultural backgrounds, as well as representation of non-binary genders [48]. Moreover, attrition analysis indicated that the responders at T4 were more frequently female than were the non-responders, and fewer responders had a non-Norwegian ethnicity. The upper middle class was overrepresented in SES, while workers were underrepresented. Regarding outliers, we have not removed any outlying observations, and the analyses did not give any indications of such. Secondly, the literature on protective factors is scarce, this study investigated potential protective factors based on a priori knowledge and available data. The majority of research investigating protective factors for suicidality have mostly derived from high-income countries, thereby underrepresenting others. Some protective factors were not included in the analysis due to low internal consistency in scales (i.e. Distractive Coping). Therefore, several protective factors remain unexplored, and possibly more could be identified. Moreover, in the current dataset, there were relatively few variables representing environmental protective factors. It is important to include a greater variety of such factors in future studies. Third, three of the protective factors were measured using single-item measures (physical activity, and school connectedness/wellbeing). Hence, these results should be interpreted and generalized with caution. Last, the current study used data (i.e., the measure of suicidal ideation) with 12 years between the measurement points. Using longitudinal measurement invariance testing (Mplus), we tested whether the parameters of the single-factor model were equal over the two timepoints. The results (data not shown) indicated acceptable fit indices for configural invariance (same factor structure across timepoints).

### Future directions

A wide range of protective factors are relevant to universal suicide prevention and can be incorporated into community settings (school-based programs, extracurricular activities) and with individuals and families. Using protective factors in early interventions, such as the school-based program “Youth Aware of Mental Health” (YAM) can offer potentially life-saving knowledge to adolescent populations about suicide and help shift focus to protective factors by reducing stigma, growing solidarity and increasing confidence [70]. This may enhance protective factors of self-concept and better coping strategies, as well as increase school wellbeing and connectedness. In clinical practice, focusing on protective factors in risk assessments can tailor treatment which motivates and increases hope [71]. Greater emphasis on protective factors can assist clinicians in preventing suicidality longitudinally along with managing risks. Potential interactions between protective factors across individual, social, and environmental levels should be explored, as well as whether there are protective factors distinctive to males and females and among suicide attempters. Research on longitudinal transitions between adolescence and adulthood that combines quantitative and qualitative knowledge is needed to provide more context about these differences.

## Conclusions

Less emotion-orientated coping, positive self-perceptions, physical activity and school wellbeing/connectedness were protective factors for SI for both sexes in this study. Attachment and family functioning, however, were protective factors for females only. Adolescent protective factors also had a longitudinal effect on SI into adulthood. This study has contributed to the understanding of protective factors for SI from an ecological and longitudinal perspective and highlighted important sex differences.

## Supplementary Information

Below is the link to the electronic supplementary material.

Supplementary file1 (DOCX 14 KB)
