# Supplementary material for: Protective factors for suicidal ideation: a prospective study from adolescence to adulthood
Source: Eur Child Adolesc Psychiatry. 2024 Feb 14;33(9):3079–89. doi: 10.1007/s00787-024-02379-w (PMC11424721; doi:10.1007/s00787-024-02379-w)
Supplement: Supplementary file 1 — Supplementary file1 (DOCX 14 KB) [file 787_2024_2379_MOESM1_ESM.docx]

**Self-reported suicidal ideations in females and males, adolescence and young adulthood**

|  |  | Adolescence (T2), mean age age = 14.9 years (SD=0.6) | |  | Young adulthood (T4), mean age = 27.2 years (SD=0.6) | |  |
| --- | --- | --- | --- | --- | --- | --- | --- |
|  |  | n ^a^ | Mean (SD) |  | n | Mean (SD) |  |
| Suicidal ideation ^a^ | | |  |  |  |  |  |
| Females | | 1222 | 0.19 (0.39) |  | 694 | 0.09 (0.30) |  |
| Males | | 1201 | 0.09 (0.27) |  | 504 | 0.12 (0.31) |  |

^a^ Measured by a suicidal ideation composite scale based on the Mood and Feelings Questionnaire (MFQ). Range 0 – 2 (mean score), a high score indicated high levels of suicidal ideations
